# Protective Effect of Djulis (*Chenopodium formosanum*) Extract against UV- and AGEs-Induced Skin Aging via Alleviating Oxidative Stress and Collagen Degradation

**DOI:** 10.3390/molecules27072332

**Published:** 2022-04-04

**Authors:** Jia-Ling Lyu, Yi-Jung Liu, Kuo-Ching Wen, Chen-Yuan Chiu, Yung-Hsiang Lin, Hsiu-Mei Chiang

**Affiliations:** 1 Ph.D. Program for Biotechnology Industry, College of Life Sciences, China Medical University, Taichung 404, Taiwan; u105306601@cmu.edu.tw (J.-L.L.); u105301602@cmu.edu.tw (Y.-J.L.); 2 Department of Cosmeceutics, College of Pharmacy, China Medical University, Taichung 404, Taiwan; kcwen0520@mail.cmu.edu.tw; 3 Institute of New Drug Development, College of Medicine, China Medical University, Taichung 404, Taiwan; 4 Department of Biological Science and Technology, College of Life Sciences, China Medical University, Taichung 404, Taiwan; 5 Center of Consultation, Center for Drug Evaluation, Taipei 115, Taiwan; kidchiou@gmail.com; 6 Research and Design Center, TCI Co., Ltd., Taipei 114, Taiwan; vincent@tci-bio.com

**Keywords:** *Chenopodium formosanum*, Djulis, ultraviolet radiation, advanced glycation end products, glycation stress, reactive oxygen species

## Abstract

Skin aging is a complex process involving photoaging and glycation stress, which share some fundamental pathways and have common mediators. They can cause skin damage and collagen degradation by inducing oxidative stress and the accumulation of reactive oxygen species (ROS). *Chenopodium formosanum* (CF), also known as Djulis, is a traditional cereal in Taiwan. This study investigated the protection mechanisms of CF extract against ultraviolet (UV) radiation and advanced glycation end products (AGEs)-induced stress. The results indicated that CF extract had strong antioxidant and free radical scavenging effects. It could reduce UV-induced intracellular ROS generation and initiate the antioxidant defense system by activating the nuclear factor erythroid 2-related factor 2 (Nrf2)/heme oxygenase-1 (HO-1) signaling pathway in human skin fibroblasts. CF extract modulated mitogen-activated protein kinase (MAPK) and transformed growth factor-beta (TGF-β) signaling pathways to alleviate oxidative stress-induced skin aging. Moreover, the results revealed that CF extract not only promoted collagen synthesis but also improved aging-induced collagen degradation. CF extract attenuated AGEs-induced ROS production and the upregulation of receptor for AGEs (RAGE). The overall results suggest that CF extract provides an effective anti-aging strategy by preventing skin damage from oxidative stress and collagen loss with potent antioxidant, anti-photoaging, and antiglycation activities.

## 1. Introduction

Aging is defined as the result of a progressive decline in the physiological function of cells and tissues of the body. During aging, the accumulation of damaged products gradually affects the organization and repair capacity of tissue. Skin, being the protective barrier for the body, is often subjected to intrinsic and extrinsic factors of aging [1]. The predominant external factor that contributes to skin aging is exposure to UV radiation in sunlight, termed photoaging [2]. Besides environmental stimuli, AGEs accumulation is considered to be an internal factor of chronological aging that triggers the skin aging process through glycation stress [3].

Both environment ROS produced by UV light and endogenous ROS formed by oxidative metabolism regulate cellular oxidative stress and destroy the collagen-rich extracellular matrix (ECM), which is not only the hallmark of skin connective tissue aging but also the major cause of skin deterioration [4]. Damage to collagen cross-links weakens the dermal structural integrity and increases the stiffness and brittleness of the collagen network, thereby promoting wrinkle formation, loss of elasticity, and dryness of the skin [5].

UVB rays are the most damaging waveband of solar radiation reaching the earth, which results in oxidative stress by stimulating ROS formation and is involved in hyperpigmentation, skin inflammation, immunosuppression, photoaging, and carcinogenesis [6,7]. It is well-known that Nrf2 acts as a major regulator of cellular antioxidant defense against cutaneous photodamage mediated by UV radiation. Cells can eliminate oxidative stress and prevent injury by regulating the Nrf2/Kelch-like ECH-associated protein 1 (Keap1) signaling pathway [8,9]. After UV exposure, excessive ROS production activates MAPK and nuclear factor-kappa B (NF-κB), which, in turn, induces the upregulation of matrix metallopeptidases (MMPs), culminating in the degradation of collagen and elastin. In addition, activator protein 1 (AP-1) inhibits TGF-β signaling and leads to a reduction in procollagen synthesis [10,11].

AGEs are a heterogeneous group of molecules formed as a result of glycation, a spontaneous non-enzymatic reaction between the reducing sugars and amino groups of proteins [3]. The interaction between AGEs and a specific receptor of AGEs (RAGE) triggers the generation of ROS and alters gene expression in AGEs-related disorders, such as diabetes and renal failure [12,13]. AGEs accumulate in ECM proteins upon aging, and N^ε^-(1-Carboxymethyl)-L-lysine (CML) produced by oxidative modification is the major glycated protein in humans [14,15]. During intrinsic aging, AGEs are expressed in the epidermis and dermis, and significantly increased AGEs accumulation in the sun-exposed areas of the skin can be observed [16]. Additionally, UV enhances the deposition of AGEs via glycation of the elastic fiber network [17].

*Chenopodium formosanum* (CF), also known as Djulis, is an edible plant in Taiwan and is usually cooked with rice or millet as a staple food. CF is considered a high nutritional value food and a source of bioactive compounds to human health [18]. Besides abundant dietary fiber, starch, grain-limited essential amino acids, and polyphenols [19,20], CF also contains a class of compounds with a long history of medicinal use, called phytoecdysteroids [21,22,23,24]. Previous studies have reported that CF displayed several biological activities, including, anti-obesity [21], antihypertensive [22], antihyperglycemic [25,26], hepatoprotective [27,28,29], anticarcinogenic [23,24,30,31] and anti-aging properties [19,32]. Nevertheless, neither the detailed skin protective effects of CF extract nor the underlying mechanisms against skin intrinsic and extrinsic aging associated with oxidative and glycation stress have been revealed to date. The purpose of this study was to clarify the antioxidant effects of CF extract and investigate the underlying anti-aging defense mechanisms of CF extract against collagen degradation by UV and AGEs in human skin fibroblasts.

## 2. Results

### 2.1. HPLC-ESI-MS/MS and MRM Quantitative Analysis

The content analysis of target bioactive compounds in CF extract was conducted by high-performance liquid chromatography (HPLC)-Mass/MS and the multiple reaction monitoring (MRM) method. The MRM ion chromatogram and mass spectrum on electrospray ionization (ESI) positive mode of 20-hydroxyecdysone and rutin standard were shown in Supplementary Materials. The transition from the precursor ion at *m*/*z* 481 to the product ion at *m*/*z* 371 for 20-hydroxyecdysone (Figure S1a) and the transition from the precursor ion at *m*/*z* 611 to the product ion at *m*/*z* 303 for rutin (Figure S1b) are shown. Figure S2 showed the total ion chromatogram (TIC) and extracted ion chromatogram (EIC) of 20-hydroxyecdysone (*m*/*z* 481) and rutin (*m*/*z* 611) in CF extract. The content of rutin and 20-hydroxyecdysone in CF extract were 14.5 ± 0.4 mg/g and 19.5 ± 0.6 mg/g, respectively.

### 2.2. Antioxidant Activity and Free Radical Scavenging Effect of CF Extract

#### 2.2.1. DPPH Free Radical Scavenging Activity

**Figure 1:**
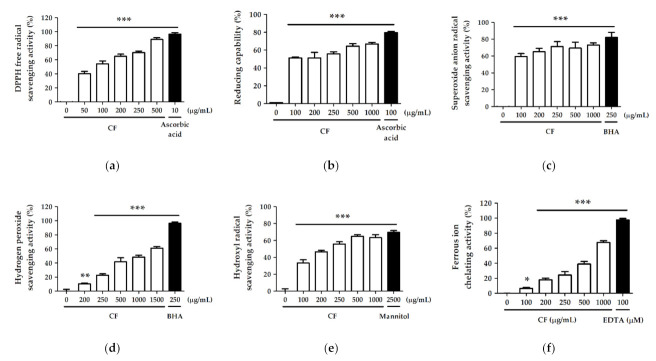
(**a**) DPPH free radical scavenging activity (%); (**b**) reducing capability (%); (**c**) superoxide anion radical scavenging activity (%); (**d**) hydrogen peroxide scavenging activity (%); (**e**) hydroxyl radical scavenging activity (%) and (**f**) ferrous ion chelating activity (%) of CF extract. Significant difference versus control group: *, *p* < 0.05; **, *p* < 0.01; ***, *p* < 0.001.

DPPH is a commonly used reagent to estimate the total free radical scavenging activity of antioxidants by detecting the ability of extracts to provide hydrogen protons. The result showed that CF extract exhibited strong scavenging activity of 40.2% ± 3.3% at 50 µg/mL and 89.1% ± 2.6% at 500 µg/mL, while the scavenging activity of ascorbic acid was 96.6% ± 2.3% at 10 µg/mL (Figure 1a). The half maximal inhibitory concentration (IC_50_) of CF extract on DPPH free radical scavenging was 84.7 ± 13.0 µg/mL.

#### 2.2.2. Reducing Capability

The reducing capability of the CF extract observed in the present study is shown in Figure 1b. The reducing capacity of CF extract was 51.3% ± 1.0% at 100 µg/mL and 66.9% ± 1.7% at 1000 µg/mL. The reducing capacity of the comparative control ascorbic acid (100 µg/mL) was 79.7% ± 1.2%.

#### 2.2.3. Superoxide Anion Radical (O_2_^−^) Scavenging Activity

The superoxide anion radical scavenging activity was 82.2% ± 6.1% for 250 µg/mL BHA (comparative control) and ranged from 59.6% ± 3.5% to 73.1% ± 2.6% for 100–1000 µg/mL CF extract (Figure 1c).

#### 2.2.4. Hydrogen Peroxide (H_2_O_2_) Scavenging Activity

The hydrogen peroxide scavenging activity ranged from 10.3% ± 1.1% to 61.1% ± 2.3% for 200–1500 µg/mL CF extract and was 96.3% ± 2.0% for BHA (250 µg/mL) (Figure 1d). The IC_50_ of CF extract was 956.5 ± 59.2 µg/mL for hydrogen peroxide scavenging activity.

#### 2.2.5. Hydroxyl Radical (· OH) Scavenging Activity

The hydroxyl radical scavenging activities of CF extract (100–1000 µg/mL) and the comparative control mannitol (µg/mL) are shown in Figure 1e. The hydroxyl radical scavenging activity of 1000 µg/mL CF extract was 63.3% ± 3.5%, and that of mannitol (2500 µg/mL) was 69.7% ± 2.2%. The scavenging effect of the CF extract was similar to that of the comparative control, and the IC_50_ of CF for hydroxyl radical scavenging activity was 240.7 ± 24.3 µg/mL.

#### 2.2.6. Ferrous Ion (Fe^2+^) Chelating Activity

Figure 1f showed the metal chelating activity of the CF extract and the comparative control EDTA. The activity ranged from 6.5% ± 1.4% to 67.6% ± 2.5% for 100–1000 µg/mL CF extract, and 97.5% ± 2.0% for 100 µM EDTA. The IC_50_ of CF extract was 625.5 ± 46.0 µg/mL for metal chelation.

### 2.3. CF Extract Alleviated UV-Induced Cytotoxicity in Hs68 Cells

**Figure 2:**
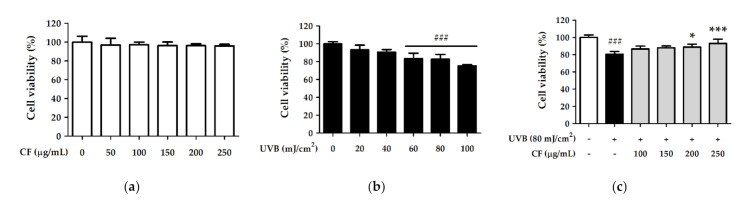
Cell viability (%) of human skin fibroblasts treated with (**a**) a series of concentrations of CF extract; (**b**) various UVB doses and (**c**) CF extract on UV radiation. Significant difference versus non-irradiated group: ###, *p* < 0.001. Significant difference versus UVB-irradiated group: *, *p* < 0.05; ***, *p* < 0.001.

To evaluate the cytotoxic effect of CF extract in human skin fibroblasts (Hs68 cells), the viability of cells treated with CF extract was assessed by MTT assay. As shown in Figure 2a, the CF extract did not induce cytotoxicity in Hs68 cells in the concentration range of 50–250 µg/mL; thus, these concentrations were chosen for the following experiments.

Hs68 cells were exposed to UVB radiation, and the cell viability was determined to evaluate the protective effect of CF extract against photodamage. The results showed that UVB (20–100 mJ/cm^2^) radiation induced cytotoxicity in Hs68 cells in a dose-dependent manner (Figure 2b). Nevertheless, Hs68 cells were exposed to UVB (80 mJ/cm^2^) and subsequently treated with CF extract (100, 150, 200, and 250 µg/mL) for 24 h; CF extract significantly alleviated UV-induced cytotoxicity (Figure 2c).

### 2.4. CF Extract Reduced UV-Induced Intracellular ROS Generation in Hs68 Cells

**Figure 3:**
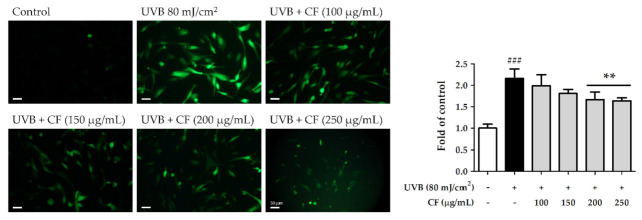
The effects of CF extract on UVB-induced intracellular ROS generation in human skin fibroblasts (scale bar = 50 µm). Significant difference versus non-irradiated group: ###, *p* < 0.001. Significant difference versus UVB-irradiated group: **, *p* < 0.01.

ROS can induce skin photoaging-related protein and downstream signal transduction by causing intracellular oxidative stress in human skin. A fluorogenic dye measures the amount of oxidative free radicals within the cells. After radiation with UVB (80 mJ/cm^2^) for 2 h, ROS production in Hs68 cells was induced 2.16-fold compared with that in the non-irradiated group. However, treatment with 100–250 µg/mL CF extract significantly decreased ROS generation. ROS were downregulated 1.64-fold compared with the radiation group by CF extract at 250 µg/mL (Figure 3).

### 2.5. CF Extract Initiated the Antioxidant Defense System by Activating the Nrf2/HO-1 Signaling Pathway in Hs68 Cells

#### 2.5.1. The Effects of CF Extract on UV-Induced Expression of Nrf2/HO-1 

**Figure 4:**
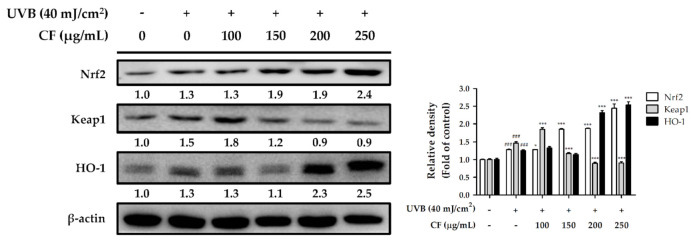
The effects of CF extract on UVB-induced Nrf2, Keap1 and HO-1 expression in human skin fibroblasts. Significant difference versus non-irradiated group: ###, *p* < 0.001. Significant difference versus UVB-irradiated group: *, *p* < 0.05; ***, *p* < 0.001.

Under normal conditions, Keap1 forms a complex with Nrf2 and promotes the ubiquitination and degradation of Nrf2. However, the Nrf2 signaling pathway is initiated when cells are stimulated by environmental oxidative stress. In this study, Hs68 cells were treated with CF extract to observe its effects on the Nrf2 signaling pathway after UV radiation. As shown in Figure 4, UVB (40 mJ/cm^2^) radiation induced Nrf2 expression and decreased Keap1 expression, whereas CF extract (250 µg/mL) significantly induced Nrf2 expression by 2.4-fold compared with the radiation group. After UV radiation and treatment with CF extract at 250 µg/mL the protein expression of HO-1 increased by 2.5-fold. The results indicated that CF extract could initiate the antioxidant defense mechanism through the Nrf2/HO-1 signaling pathway and protect cells from oxidative stress in a biological system.

#### 2.5.2. The Effects of CF Extract on UV-Induced Nuclear Translocation of Nrf2

**Figure 5:**
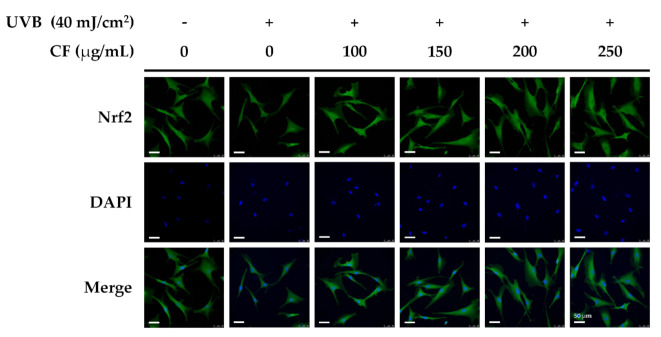
The effects of CF extract on UVB-induced nuclear translocation of Nrf2 in human skin fibroblasts (scale bar = 50 µm).

UV radiation promotes Nrf2 activation and translocation into the nucleus of cells. In this study, the Nrf2 immunofluorescence staining assay in human skin fibroblasts was performed to determine the level of Nrf2 activation. The translocation of Nrf2 from the cytoplasm to the nucleus increased after UVB radiation, whereas the translocation was also enhanced after CF extract treatment (Figure 5). The results of the immunofluorescence staining assay were consistent with Nrf2 expression in Figure 4.

### 2.6. CF Extract Modulated Skin Photoaging-Associated Cellular Signaling Pathway in Hs68 Cells

#### 2.6.1. The Effects of CF Extract on UV-Induced Expression of MMP-1, -3, -9 and TIMP-1

**Figure 6:**
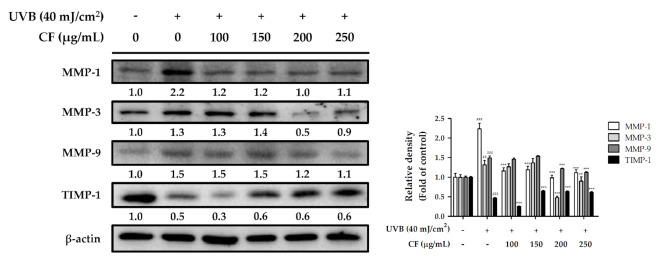
The effects of CF extract on UVB-induced MMP-1, -3, -9 and TIMP-1 expression in human skin fibroblasts. Significant difference versus non-irradiated group: ##, *p* < 0.01; ###, *p* < 0.001. Significant difference versus UVB-irradiated group: **, *p* < 0.01; ***, *p* < 0.001.

UV radiation induces the phosphorylation of MAPK, which triggers downstream signal transduction to activate transcription factor AP-1 and enhance MMP expression. When the skin is exposed to oxidative stress, MMPs are upregulated, which then induce collagen degradation and wrinkle formation. As shown in Figure 6, UVB radiation significantly elevated MMP-1, -3, and -9 expression (2.2-fold, 1.3-fold, and 1.5-fold, respectively, compared with the control group), whereas CF extract attenuated the expression of MMPs. CF extract treatment at 250 µg/mL significantly suppressed the levels of UVB-induced expression of MMP-1, MMP-3, and MMP-9 by 1.1-fold, 0.9-fold, and 1.1-fold, respectively, compared with the control group.

Tissue inhibitors of metalloproteinases (TIMPs) are inhibitors of MMPs expressed in the skin connective tissue. TIMP-1 expression was decreased after UVB radiation and enhanced by CF extract (100–250 µg/mL) (Figure 6). These results demonstrated that CF extract treatment prevented the UV-induced elevation of MMP-1, -3, and -9 levels and reduction of TIMP-1; therefore, CF extract may decrease UV-induced skin extracellular matrix breakdown and maintain structural integrity in the dermis.

#### 2.6.2. The Effects of CF Extract on UV-Induced Expression of AP-1 and MAPK

**Figure 7:**
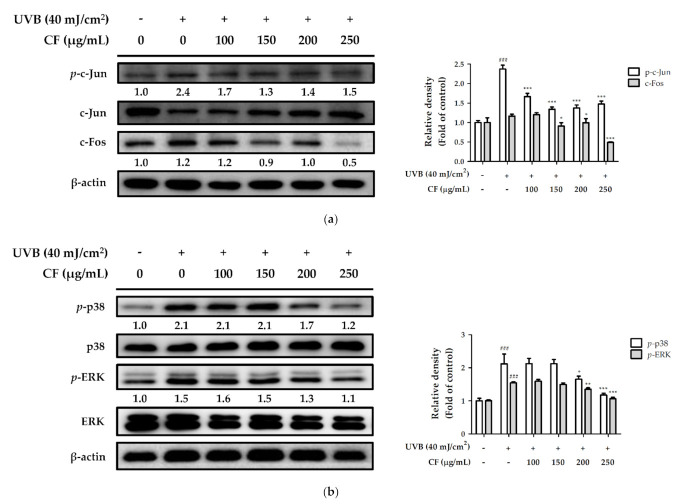
The effects of CF extract on UVB-induced expression of (**a**) AP-1 and (**b**) MAPK in human skin fibroblasts. Significant difference versus non-irradiated group: ###, *p* < 0.001. Significant difference versus UVB-irradiated group: *, *p* < 0.05; **, *p* < 0.01; ***, *p* < 0.001.

UVB radiation upregulated the expression of *p*-c-Jun and c-Fos (2.4- and 1.2-fold compared with the control group), whereas they were downregulated by CF extract (100–250 µg/mL). Treatment with CF extract significantly decreased the expression of *p*-c-Jun and c-Fos at 250 µg/mL (Figure 7a). Figure 7b showed that UVB exposure increased the expression of phosphorylation of p38 and ERK to 2.1- and 1.5-fold relative to total protein levels in Hs68 cells. However, treatment with CF extract (100–250 µg/mL) significantly inhibited the UVB-induced MAPK overexpression. The phosphorylation of p38 and ERK was suppressed to basal levels with a 250 µg/mL concentration of CF extract. Moreover, CF extract could inhibit UV-induced overexpression of AP-1, protecting the skin from photoaging.

#### 2.6.3. The Effects of CF Extract on UV-Inhibited Expression of TGF-β and Smad3

**Figure 8:**
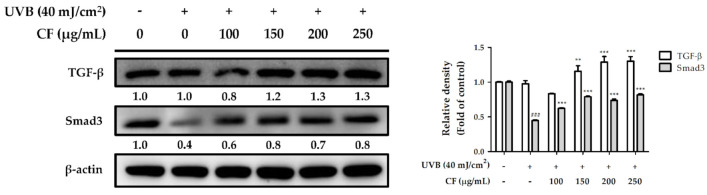
The effects of CF extract on UVB-inhibited TGF-β and Smad3 expression in human skin fibroblasts. Significant difference versus non-irradiated group: ###, *p* < 0.001. Significant difference versus UVB-irradiated group: **, *p* < 0.01; ***, *p* < 0.001.

TGF-β plays a central role in extracellular matrix synthesis and controls collagen homeostasis in the human dermis. Increased ROS in fibroblasts impairs TGF-β signaling by downregulating TGF-β and Smad3 expression, which leads to the loss of collagen and destruction of dermal tissue in aged skin. Similarly, in our study, UVB radiation downregulated the expression of TGF-β and Smad3 to inhibit collagen synthesis. Compared with the control group, TGF-β and Smad3 expression were lower in the UVB-irradiated group; however, CF extract (100–250 µg/mL) significantly restored protein expression (Figure 8).

### 2.7. CF Extract Attenuated AGEs-Induced ROS Production and RAGE Expression in Hs68 Cells

**Figure 9:**
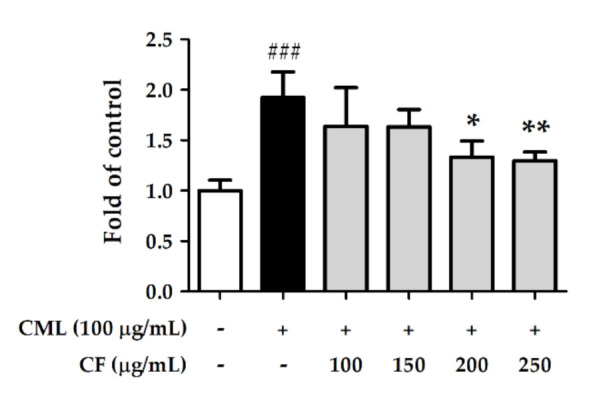
The effects of CF extract on CML-treated intracellular ROS generation in human skin fibroblasts. Significant difference versus non-treated group: ###, *p* < 0.001. Significant difference versus CML-treated group: *, *p* < 0.05; **, *p* < 0.01.

**Figure 10:**
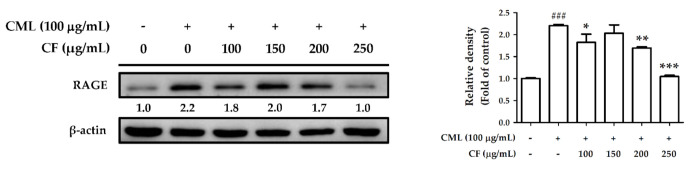
The effects of CF extract on CML-treated RAGE expression in human skin fibroblasts. Significant difference versus non-treated group: ###, *p* < 0.001. Significant difference versus CML-treated group: *, *p* < 0.05; **, *p* < 0.01; ***, *p* < 0.001.

To investigate the effect of CF extract on glycation stress, Hs68 cells were treated with CML. After treating the cells with CML (100 µg/mL) for 6 h, the amount of ROS generated was 1.92-fold higher than that in the non-treated group. However, treatment with CF extract (100–250 µg/mL) significantly decreased ROS production. ROS was downregulated 1.30-fold compared with the CML-treated group by CF extract at 250 µg/mL (Figure 9). Furthermore, CML (100 µg/mL) significantly increased RAGE expression to 2.2-fold compared with the control group, but it was attenuated by treatment with CF extract (Figure 10), indicating that CF extract reduced the induction mainly caused by CML.

### 2.8. CF Extract Promoted Collagen Synthesis and Improved Aging-Induced Collagen Degradation in Hs68 Cells

#### 2.8.1. The Effects of CF Extract on Collagen Synthesis

**Figure 11:**
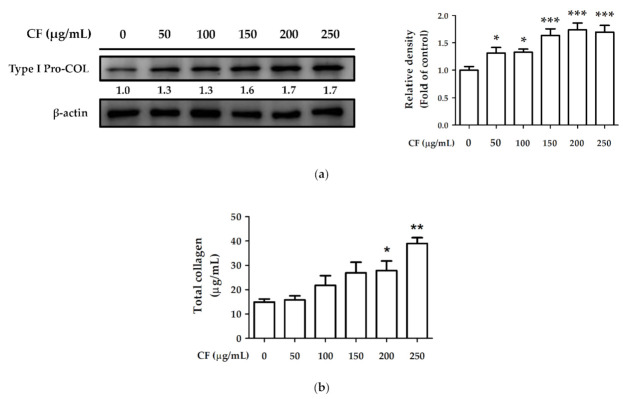
The effects of CF extract on (**a**) type I pro-collagen expression and (**b**) total collagen content in human skin fibroblasts. Significant difference versus non-treated group: *, *p* < 0.05; **, *p* < 0.01; ***, *p* < 0.001.

Hs68 cells were treated with CF extract (50–250 µg/mL) for 24 h, and type I procollagen expression was measured using western blotting. After treatment with CF extract, type I procollagen expression was significantly upregulated (1.7-fold compared with the control group) (Figure 11a). The total collagen content in the control group was 14.93 ± 1.27 µg/mL which increased to 39.00 ± 2.39 µg/mL after treatment with 250 µg/mL CF extract (Figure 11b). The results of type I procollagen expression and total collagen content indicated that CF extract promoted collagen synthesis and production.

#### 2.8.2. The Effects of CF Extract on UV and AGEs-Induced Collagen Degradation

**Figure 12:**
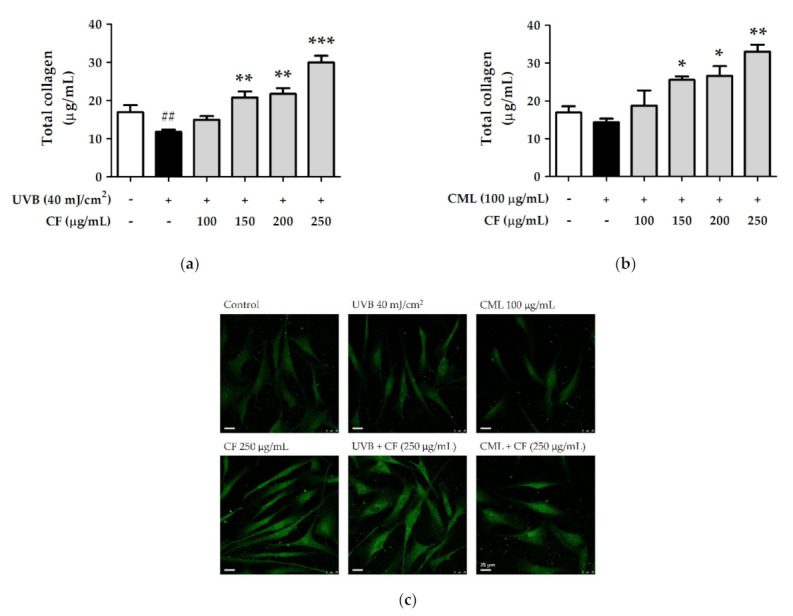
The effects of CF extract on total collagen content of (**a**) UVB-irradiated and (**b**) CML-treated groups. (**c**) Immunofluorescence staining of collagen in human skin fibroblasts (scale bar = 25 µm). Significant difference versus control group: ##, *p* < 0.01. Significant difference versus UVB-irradiated or CML-treated group: *, *p* < 0.05; **, *p* < 0.01; ***, *p* < 0.001.

Hs68 cells exposed to UVB (40 mJ/cm^2^) radiation or treated with CML (100 µg/mL) were treated with CF extract for 24 h. As shown in Figure 12a, the total collagen content was significantly decreased after UVB radiation, and treatment with CF extract restored collagen and significantly increased collagen formation at 250 µg/mL. Similar results were shown in collagen production treated with CML and a series of CF extracts. CF extract attenuated the CML-treated reduction in total collagen (Figure 12b).

The immunofluorescence staining assay was used to determine the collagen level in fibroblast cells (Figure 12c). Both UVB radiation and CML treatment reduced collagen content, while CF extract improved collagen degradation. The results of the immunofluorescence staining assay of CF extract on collagen were consistent with the collagen content in Figure 11b and Figure 12a,b.

## 3. Discussion

Fibroblasts play a crucial role in the turnover of the dermal extracellular matrix, including the synthesis of collagen, elastic fiber, and glycosaminoglycan to form dense connective tissue [33]. The accumulation of UV and AGEs stress causes collagen degradation and interferes with each other during intrinsic and extrinsic aging [3,34]. The development of natural products or materials with antioxidant, anti-photoaging, and antiglycation activities for the amelioration of skin aging have been ongoing in recent years.

*Chenopodium formosanum* (CF) is a native grain plant grown in Taiwan, mainly cultivated in aboriginal residential areas. Apart from cereal use, the application of CF based on antioxidant properties has attracted increasing attention [28,29,30,35]. Polyphenols are well-known antioxidants and skin protectors by possessing strong free radical scavenging activity [36], reducing inflammation and absorbing UV radiation to provide skin photoprotection [37,38]. Rutin is a flavonoid widely distributed in fruits and vegetables and the representative polyphenol in CF indicated by previous studies [21,22]; several reports have demonstrated the biological effects of rutin on ROS-induced skin aging [39] and suggested that rutin effectively inhibits the formation of AGEs on collagen synthesis [40]. CF has been indicated to protect skin against UV-induced epidermal damage and inflammation in keratinocytes and mouse models, and rutin is the major component in CF that contributed to the protective effect [19]. In this study, the results showed that HaCaT cells treatment with CF had a higher survival rate and less production of interleukin-6, MMP-1 and ROS in UVB-irradiated conditions. Previous study used keratinocytes exposed to UVB radiation to mimic a skin photoaging model, and the fibroblasts were used in the present study to explore the mechanisms and effects of intrinsic factors (AGEs) and extrinsic factors (UV) on dermis skin aging. The results of this study showed that CF decreased UV-induced cytotoxicity and provided a good ROS scavenging activity, and the results were consistent with Hong’s study [19]. This study considered more factors of skin aging and investigate the anti-glycation effect of CF on intrinsic aging, and the findings further confirmed that CF has an excellent skin protection efficacy via alleviating oxidative stress and collagen degradation.

20-Hydroxyecdysone is a bioactive compound belonging to phytoecdysteroids, commonly found in *Chenopodium formosanum* [21,22,23,24]. *Microsorum grossum* (Polypodiaceae) is one of the most commonly used ferns in Polynesian traditional medicine, and its frond and rhizome extracts contain 20-hydroxyecdysone as the main bioactive components. *Microsorum grossum* was known to have UVB-protective effects on human dermal fibroblasts by upregulating the antioxidant enzyme HO-1 and inhibiting stress-induced premature senescence [41]. *Chenopodium quinoa* (Amaranthaceae) is the seed crop known as the “golden grain” by the native Andean people in South America, including 20-hydroxyecdysone, which has been shown to inhibit intracellular ROS production and MMPs activity [42]. In addition, 20-hydroxyecdysone isolated from *C. quinoa* seeds possess strong inhibition activity against collagenase and the DPPH free radicals, and a potent ability to chelate iron ions [43]. Many previous studies have reported that CF extract contains rutin and 20-hydroxyecdysone as its rich bioactive ingredient, other plants containing these compounds also possess similar activities and skin care potential. The primary advantage of botanicals is their complex composition and the synergistic effect of related compounds with multiple activities to obtain greater efficacy [44]. Plant extracts pose a promising future in skin care due to their appeal as natural products, perception as safe, and abundance and sustainability.

ROS are byproducts of regular oxidative cell function in the electron transport chain of aerobic metabolism reaction. Excessive UV exposure and other environmental stressors overwhelm the cutaneous antioxidant capacity further inducing ROS production, DNA fragmentation, lipid peroxidation, and apoptosis [45,46]. High levels of ROS initiate aging signal cascades in skin cells, thereby promoting cell senescence, even death [47]. According to the results of cell assay, CF extract with no cytotoxic effects on fibroblasts significantly rescued UV-induced cell cytotoxicity and showed good photoprotective activity. Furthermore, ROS formed in fibroblasts was significantly increased either by UV radiation or by AGEs treatment and both were effectively inhibited by CF extract. The strong radical scavenging activity of CF was also verified by different antioxidant assays. Polyphenols are capable of donating electrons to free radicals and converting them into more stable non-reactive species owing to the presence of hydroxyl groups in the ring structure [48,49]. CF rich in polyphenols may prevent ROS-induced skin injury by terminating the free radicals chain reaction. The multiple antioxidant-related compounds in CF extract provide a synergistic effect to enhance the overall activity of CF extract and make CF extract a strong antioxidant to protect skin from oxidative stress.

The skin possesses an endogenous antioxidant defense system to deal with oxidative stress induced by aging. Nrf2 is a transcription factor that acts against oxidative stress, and Keap1 is a pressure sensor that regulates Nrf2 expression [8]. When the intracellular pressure imbalance causes the configuration change of Keap1 and loses its ability to bind to Nrf2, Nrf2 translocates into the nucleus and enhances the level of intrinsic antioxidant enzymes to prevent oxidative damage and exert photoprotective activity [50,51]. The results demonstrated that CF extract could initiate the Nrf2 signaling pathway and antioxidant defense system when cells were stimulated by UV-induced oxidative stress. CF extract upregulated the expression of Nrf2 and HO-1. The cellular defensive properties of CF extracts can be explained by their ability to either directly neutralize ROS or indirectly upregulate the expression of cellular defensive proteins. Based on the above results, CF extract can reduce intracellular oxidative stress and be used as a photo protectant. In addition to its direct effect on ROS generation, CF extract also protected fibroblasts against UV damage by enhancing intracellular antioxidant defense mechanisms via Nrf2 and its target factor, HO-1.

Photoaging is caused by an imbalance between the accumulation and degradation of ECM after repetitive UV absorption. UV radiation damages alternations of cutaneous collagen and hastens the generation of ROS, which in turn act on cells and matrix components to mediate further turnover [33,47]. ROS and oxidative stress result in damaged skin via activation of AP-1 signaling and elevation of multiple MMPs. During normal tissue maintenance, collagen undergoes continuous turnover. However, UV radiation is known to induce three different MMPs, collagenase (MMP-1), stromelysin (MMP-3), and gelatinase (MMP-9), to break collagen and elastic fibers, resulting in fragmentation and disorganization [52,53]. TIMPs are inhibitors of MMPs, and the homeostasis between these two proteins plays an important role in the functional and structural integrity of the ECM [54,55,56]. In this study, CF extract inhibited UVB-induced AP-1 and phosphorylation of ERK and p38 proteins in human skin fibroblasts. Furthermore, treatment with CF extract blocked UVB-induced collagen degradation by inhibiting MMP-1, -3, and -9 expression in human skin fibroblasts and inducing TIMP-1 expression against MMPs activity. The TGF-β pathway is the major pathway regulating procollagen biosynthesis and ROS production [57]. Oxidative stress inhibits TGF-β signaling by downregulating Smad3, which contributes to the loss of collagen content in aged skin [4]. The results showed that CF extract increased TGF-β and Smad3 expression to enhance the total collagen content. Based on the above results, CF extract ameliorated UVB-induced collagen degradation through ROS scavenging, MMPs inhibition, and TGF-β upregulation.

One of the factors of intrinsic skin aging is the progressive accumulation of AGEs throughout the lifespan. RAGE belongs to the immunoglobulin superfamily of cell surface receptors, which interacts with several ligands, especially CML, one of the glycation products of the Maillard reaction [58]. RAGE is highly expressed in skin keratinocytes and fibroblasts by interaction with AGEs followed by UVB radiation [59] and upregulated in sun-exposed skin [60]. UV-induced oxidation accelerates the formation of human skin elastin in actinic elastosis, and AGEs accumulate on skin collagen and elastin, which interfere with normal skin function [61]. Activation of RAGE decreases type I collagen synthesis and matrix production in fibroblasts [62]. A previous study mentioned that CF extract inhibited the formation of AGEs in the cell-free system [32], but the detailed antiglycation mechanism in dermal fibroblasts remains unclear. Based on the results in this study, we presumed that preventing collagen degradation by blocking AGE–RAGE interaction and decreasing ROS production inside the cells are the possible antiglycation pathways of CF extract.

Glycation-induced biological products are known to be associated with skin aging, diabetes, and the progression of some tumors. During the aging process, the accumulation of AGEs changes the mechanical properties of skin by increasing stiffness [34]. Therefore, the presence of AGEs is considered to be one of the factors for delayed wound healing and loss of elasticity of the healed skin. The cross-talk between RAGE and TGF-β1 affects wound healing in diabetes by regulating collagen turnover and cytokine production in AGEs-treated fibroblast cells [63]. In this study, CF extract not only promotes pro-collagen synthesis in fibroblasts but also improves the degradation of collagen caused by intrinsic and extrinsic skin aging. Moreover, CF extract upregulated TGF-β expression and downregulated RAGE expression. Based on the above research results, the role of CF in glycation stress-related wound healing mechanisms can be further explored in the future.

## 4. Materials and Methods

### 4.1. Materials and Chemicals

Reagents used in the cell culture, including Dulbecco’s modified Eagle’s medium (DMEM), fetal bovine serum (FBS), penicillin-streptomycin, and trypsin-EDTA, were obtained from Gibco, Thermo Fisher Scientific, Inc. (Waltham, MA, USA). CML was purchased from Cayman Chemical (Ann Arbor, MI, USA). Sodium dodecyl sulfate (SDS), 3-(4,5-Dimethyl-2-thiazolyl)-2,5-diphenyl-2H-tetrazolium bromide (MTT), and potassium phosphate-potassium hydroxide (KH_2_PO_4_-KOH) were purchased from USB Corporation (Cleveland, OH, USA). 2,2-Diphenyl-1-picrylhydrazyl (DPPH), potassium ferricyanide(III) (K_3_Fe(CN)_6_), ferric chloride (FeCl_3_), ascorbic acid, phenazine methosulfate (PMS), β-nicotinamide adenine dinucleotide, reduced disodium salt hydrate (NADH), nitrotetrazolium blue chloride (NBT), butylated hydroxyanisole (BHA), 2-deoxy-D-ribose, thiobarbituric acid (TBA), trichloroacetic acid (TCA), mannitol, ferrous chloride (FeCl_2_), ethylenediaminetetraacetic acid (EDTA), 2′, 7′-dichlorofluorescin-diacetate (H_2_DCFDA), 2-propanol and 20-hydroxyecdysone were purchased from Sigma-Aldrich (Saint Louis, MO, USA). Rutin was obtained from ACROS Organics (Morris Plains, NJ, USA). HPLC-grade acetonitrile and formic acid were obtained from J.T. Baker, Thermo Fisher Scientific, Inc. (Waltham, MA, USA). All other chemicals used were of analytical grade or higher grade.

### 4.2. Preparation of CF Extract

CF used in this study was purchased from Pingtung County, Taiwan, and the CF extract was supplied by TCI Co., Ltd. (Taipei, Taiwan). The preparation method of CF extract has been reported previously [32]. Briefly, the unhulled *Chenopodium formosanum* was added to water at the ratio of 1:10 (*w*/*v*) and extracted in three different temperatures, first at 25 °C, then at 50 °C, and finally at 70 °C. The mixture was centrifuged at 4600× *g* for 20 min and the supernatant was collected. The powder of CF extract was obtained after removing solvent from the supernatant by freeze-drying and stored at room temperature.

### 4.3. HPLC-ESI-MS/MS and MRM Quantitative Analysis

The HPLC-ESI mass spectrometric analysis was performed using Dionex Ultimate 3000 liquid chromatography (Thermo Scientific, Waltham, MA, USA) coupled with an ion trap mass spectrometer (HCT Ultra, Bruker Daltonics, Bremen, Germany). The separation method was performed as described by Chen et al., with minor modification [22]. CF extract was injected into the Atlantis T3 C18 column (2.1 mm ID × 150 mm, 3 µm particle size, Waters Corp., Milford, MA, USA), which was connected with a guard column (SecurityGuard™ C18 2.0 mm ID × 4.0 mm, Phenomenex Inc., Torrance, CA, USA). The mobile phases employed were: 0.1% formic acid in water (solvent A) and 0.1% formic acid in acetonitrile (solvent B) at a flow rate of 0.3 mL/min. A multi-step gradient elution was carried out with 5–45% B in 15 min, 45–95% B in 5 min and finally 95% B isocratic elution for 5 min.

Mass spectrometry was carried out by the program Esquire Control (V6.2) and LC/MS was controlled by Hystar (V3.2) (Bruker Daltonics, Bremen, Germany). The mass spectrometer operating parameters were set as follows: nebulizer pressure 30 psi, dry gas 12 L/min, dry temperature 300 °C, capillary voltage of 3600 V with an end plate of −500 V. MS/MS was applied to all major ions in the selected mass range. Multiple reaction monitoring (MRM)-profiling was performed by analyzing the following ion transitions: the transition from the precursor ion at *m*/*z* 481 to the product ion at *m*/*z* 371 for 20-hydroxyecdysone and the transition from the precursor ion at *m*/*z* 611 to the product ion at *m*/*z* 303 for rutin. Ion isolation width was set to 4 *m*/*z* with a fragmentation amplitude of 0.5 V. The compound was identified and quantified by comparing the mass spectra obtained by ESI-MS/MS with commercial reference standards.

### 4.4. Antioxidant Capacity Measurement

#### 4.4.1. DPPH Free Radical Scavenging Assay

Reaction mixtures containing a methanol solution of DPPH and serial CF extract dilutions of 50–500 µg/mL were placed in a 96-well microplate and incubated for 30 min. The absorbance was determined at 517 nm using a microplate reader (Sunrise, Tecan, Salzburg, Austria). Ascorbic acid (10 µg/mL) was used as a comparative control [64].

#### 4.4.2. Reducing Power Assay

Serial concentrations of CF extract (100–1000 µg/mL) were mixed with phosphate buffer and K_3_Fe(CN)_6_ and then incubated at 50 °C for 20 min. TCA was added, and the reaction mixture was centrifuged at 3000 rpm for 10 min. FeCl_3_ solution was mixed with supernatant and the absorbance was measured at 700 nm using a multi-mode reader (Synergy HTX, BioTek Instruments, Winooski, VT, USA). Ascorbic acid (100 µg/mL) was used as a comparative control [64].

#### 4.4.3. Superoxide Anion Radical (O_2_^−^) Scavenging Assay

Reaction mixtures containing PMS, NADH, and NBT were prepared in phosphate buffer and serial CF extract dilutions of 100–1000 µg/mL. The mixture was reacted for 5 min, and the absorbance was read at 560 nm using a microplate reader (Sunrise, Tecan, Salzburg, Austria). BHA (250 µg/mL) was used as a comparative control [65].

#### 4.4.4. Hydrogen Peroxide (H_2_O_2_) Scavenging Assay

The activity of CF extract to scavenge H_2_O_2_ was performed as previously described [65]. Various concentrations of CF extract (200–1500 µg/mL) were added to the H_2_O_2_ solution and reacted in the dark for 10 min. The absorbance of the reaction mixture was recorded at 230 nm using a multi-mode reader (Synergy HTX, BioTek Instruments, Winooski, VT, USA), and BHA (250 µg/mL) was used as a comparative control.

#### 4.4.5. Hydroxyl Radical (· OH) Scavenging Assay

The assay was conducted by mixing CF extract dilutions (100–1000 µg/mL), KH_2_PO_4_-KOH buffer, 2-deoxy-D-ribose, FeCl_3_, ascorbic acid, EDTA, H_2_O_2_, and deionized water. After incubation at 37 °C for 1 h, TBA and TCA were added to the mixture, and the mixture was incubated at 100 °C for 15 min and then centrifuged at 4000 rpm and 25 °C for 10 min. Subsequently, the absorbance of the supernatant was measured at 532 nm using a microplate reader (Synergy HTX, BioTek Instruments, Winooski, VT, USA). Mannitol (2500 µg/mL) was used as a comparative control [64].

#### 4.4.6. Ferrous Ion (Fe^2+^) Chelating Assay

The chelation of ferrous ions by CF extract was carried out according to the procedure described previously [65]. CF extract (100–1000 µg/mL) was mixed with FeCl_2_ solution and the reaction was initiated by adding ferrozine. After the mixture had reached equilibrium, the Fe^2+^-chelating activity of CF extract was monitored by measuring the absorbance of the Fe^2+^-ferrozine complex at 562 nm using a multi-mode reader (Synergy HTX, BioTek Instruments, Winooski, VT, USA). EDTA (100 µM) was used as a comparative control.

### 4.5. Cell Culture

Hs68 cells were purchased from the Bioresource Collection and Research Center (BCRC, Hsinchu, Taiwan). Cells were maintained in 10-cm cell culture dishes and grown in DMEM supplemented with 10% FBS, 100 U/mL penicillin, and 100 µg/mL streptomycin at 37 °C in an incubator containing 5% CO_2_. At near confluence (80–90%), cells were disaggregated in trypsin-EDTA and subcultured [55].

### 4.6. UV Exposure and AGEs Treatment

The UV exposure dose selection for Hs68 cells referred to previous studies [55,56]. Prior to treatment, cells were rinsed with PBS twice and irradiated with different doses of UVB (mJ/cm^2^). The UVB energy spectrum (280–320 nm) was supplied by UV Crosslinker CL-1000M (UVP, Upland, CA, USA) with an emission peak at 302 nm and the exposure time was 15–30 s. The UVB radiation doses in cell viability assay were 20–100 mJ/cm^2^ to evaluate the appropriate UVB exposure dose to induce cytotoxicity. Hs68 cells were radiated with a high dose UVB (80 mJ/cm^2^) for 2 h to investigate the rapid radical scavenging ability of CF extract against short-term overproduced intracellular ROS. Other experiments use UVB radiation (40 mJ/cm^2^) in Hs68 cells for 24 h to mimic cells under long-term oxidative stress conditions. CML was supplied as a crystalline solid and aqueous solutions of CML were prepared by dissolving the compound in PBS buffer. The cells were treated with 100 µg/mL CML.

### 4.7. Cell Viability Assay

Hs68 cells were seeded into 24-well plates (4 × 10^4^ cells/well), allowed to attach overnight, and were treated with 1 mL of various concentrations of CF extract (50–250 µg/mL) dissolved in DMEM for 24 h. MTT solution was added to the plates followed by incubation at 37 °C for 4 h. Then, 2-propanol was added to dissolve the MTT formazan crystals and the absorbance was measured at 570 nm using a microplate reader (Sunrise, Tecan, Salzburg, Austria) [56].

### 4.8. Measurement of Intracellular ROS

The assay is based on the use of an established non-fluorescent (H_2_DCFDA)/fluorescent (DCF) system that measures ROS activity within the cell. Hs68 cells were seeded into 24-well plates (4 × 10^4^ cells/well) and allowed to attach overnight. Cells were incubated in a serum-free medium in the presence of various concentrations of CF extract (100–250 µg/mL) after UVB radiation or CML treatment. The cells were washed with PBS, and the ROS detection reagent DCFDA was added to each well for 30 min. Images were captured using a fluorescence microscope (Leica DM IL LED, Leica Microsystems, Wetzlar, Germany), and the fluorescence (excitation/emission: 488 nm/520 nm) were measured using a microplate reader (Synergy HTX, BioTek Instruments, Winooski, VT, USA) [55].

### 4.9. Protein Expression by Western Blot Analysis

After the indicated treatments, Hs68 cells (5 × 10^5^ cells/dish) were collected and homogenized with protein extraction buffer on ice. The cell lysates were vortexed for 30 min at 4 °C and centrifuged to obtain the supernatant as an intracellular protein. Prepared samples containing equal amounts of total protein were separated by electrophoresis on SDS-polyacrylamide gels and transferred to polyvinylidene difluoride blotting membranes. Nonspecific binding was blocked with non-fat milk in TBST buffer. The membrane was incubated with primary antibodies against, c-Jun, c-Fos, MMP-1, MMP-3, TGF-β, Smad3, and pro-COL1A1 (Santa Cruz Biotechnology, Inc., Dallas, TX, USA), MMP-9, TIMP-1, and HO-1 (GeneTex, Inc., Irvine, CA, USA). Nrf2, Keap1, RAGE, *p*-c-Jun, *p*-p38, *p*-ERK and β-actin (Cell Signaling Technology, Inc., Danvers, MA, USA) at 4 °C overnight. Subsequently, the membranes were washed with TBST buffer several times, and the corresponding anti-immunoglobulin G-horseradish peroxidase was used as the secondary antibody. The blots were incubated with a chemiluminescence detection reagent. Immunoreactive bands were detected with the ECL western blotting detection system (LAS-4000, Fujifilm, Tokyo, Japan), and the signal intensity was quantified using ImageJ software (National Institutes of Health, Bethesda, MD, USA) [56].

### 4.10. Immunofluorescence Staining

The cells were seeded on slides in a 6-well plate (Hs68 cells: 1 × 10^5^ cells/well) and allowed to attach overnight. Cells were irradiated with UVB (40 mJ/cm^2^) and treated with 1 mL CF extract dissolved in DMEM. The cells were washed with PBS, fixed with paraformaldehyde for 30 min, and blocked using non-fat milk with Triton X-100/PBS-buffer (Thermo Fisher Scientific, Inc., Waltham, MA, USA) for 60 min. Samples were incubated with primary rabbit anti-Nrf2 antibody overnight at 4 °C. Slides were washed with PBS and then incubated with an anti-rabbit secondary antibody for 2 h at room temperature. Slides were mounted using ProLong^®^ Gold Antifade Mountant with DAPI (Invitrogen, Waltham, MA, USA). All samples were analyzed using a confocal spectral microscope imaging system (Leica Microsystems, Wetzlar, Germany) [51].

### 4.11. Determination of Total Collagen Content

The total collagen content was quantified using the Sirius Red Collagen Detection kit (Chondrex Inc., Redmond, WA, USA). The cell culture medium was collected and mixed with the concentration reagent. The mixture was vortexed and incubated at 4 °C for 24 h. After incubation, the mixture was centrifuged at 10,000 rpm, and the supernatant was discarded. Following this, acetic acid was added to the microtube to dissolve the pellet, and the obtained solution was used as the test sample. Sirius Red solution, for collagen staining, was added to each tube, vortexed, incubated for 20 min at room temperature, and centrifuged. The supernatant was removed by carefully pipetting without disturbing the pellet. Washing solution was added to each tube, and the above steps were repeated once. Finally, an extraction buffer was added and the absorbance was measured at 540 nm using a microplate reader (Sunrise, Tecan, Salzburg, Austria).

### 4.12. Statistical Analysis

Data are presented as mean ± standard deviation at least three independent experiments. All statistical analyses were performed using GraphPad Prism 5 (GraphPad Software Inc., San Diego, CA, USA). Significant differences between groups in the experiments were analyzed using one-way ANOVA, followed by Tukey’s post-hoc test. Differences with *p* < 0.05 were considered statistically significant.

## 5. Conclusions

**Figure 13:**
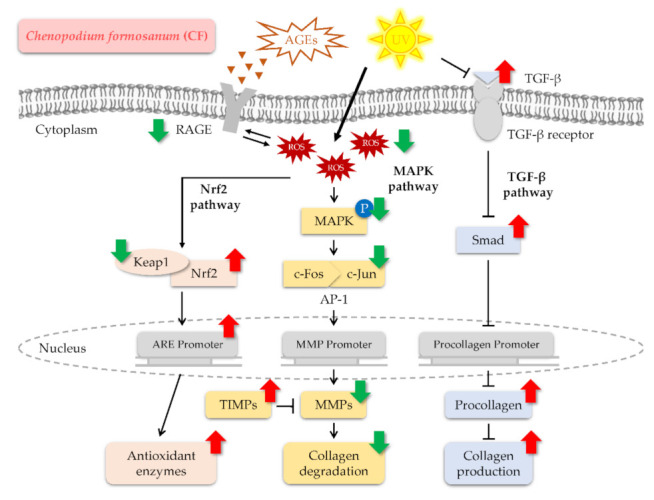
Hypothetical model of CF-mediated defense pathways against UV and AGEs-induced oxidative stress and collagen degradation in Hs68 cells. (↑, up-regulation; ↓, down-regulation).

The present study reveals the molecular mechanisms responsible for the protective effect of CF against UV and AGEs-induced intracellular ROS generation and collagen degradation in skin fibroblasts. As a consequence, our study provides current understanding and information on the role of CF extract in the cellular defense system against oxidative stress via activation of the Nrf2/HO-1 signaling pathway. Additionally, CF not only modulates MAPK/AP-1/MMPs and TGF-β signaling pathways to prevent oxidative stress-induced collagen loss but also attenuates the upregulation of receptors for AGEs. CF extract can be developed into healthy foods or applied to skincare products and cosmetics as an antioxidant, anti-photoaging, and antiglycation agent to delay aging in the future. The regulatory mechanisms of CF extract in Hs68 cells are shown in Figure 13.

## Data Availability

Not applicable.

## Supplementary Materials

The following supporting information can be downloaded at: https://www.mdpi.com/article/10.3390/molecules27072332/s1, Figure S1: The MRM ion chromatogram and mass spectrum on ESI positive mode of 20-hydroxyecdysone and rutin standard. Figure S2: The total ion chromatogram (TIC) and extracted ion chromatogram (EIC) of 20-hydroxyecdysone (*m*/*z* 481) and rutin (*m*/*z* 611) in CF extract under MRM monitoring.
