# Supplementary material for: Protective Effect of Djulis (Chenopodium formosanum) Extract against UV- and AGEs-Induced Skin Aging via Alleviating Oxidative Stress and Collagen Degradation
Source: Molecules. 2022 Apr 4;27(7):2332. doi: 10.3390/molecules27072332 (PMC9000422; doi:10.3390/molecules27072332)
Supplement: Supplementary file 1 [file molecules-27-02332-s001.zip › molecules-1656975-supplementary.pdf]

molecules

Supplementary Materials

Protective Effect of Djulis (*Chenopodium formosanum*) Extract against UV- and AGEs-Induced Skin Aging via Alleviating Oxidative Stress and Collagen Degradation

Jia-Ling Lyu [1,2,3], Yi-Jung Liu [1,2,4], Kuo-Ching Wen [2], Chen-Yuan Chiu [5], Yung-Hsiang Lin [6] and Hsiu-Mei Chiang [1,2,*]

1 Ph.D. Program for Biotechnology Industry, College of Life Sciences, China Medical University, Taichung 404, Taiwan; u105306601@cmu.edu.tw (J.-L. L.); u105301602@cmu.edu.tw (Y.-J. L.)
2 Department of Cosmeceutics, College of Pharmacy, China Medical University, Taichung 404, Taiwan; kcwen0520@mail.cmu.edu.tw
3 Institute of New Drug Development, College of Medicine, China Medical University, Taichung 404, Taiwan
4 Department of Biological Science and Technology, College of Life Sciences, China Medical University, Taichung 404, Taiwan
5 Center of Consultation, Center for Drug Evaluation, Taipei 115, Taiwan; kidchiou@gmail.com
6 Research and Design Center, TCI Co., Ltd., Taipei 114, Taiwan; vincent@tci-bio.com
* Correspondence: hmchiang@mail.cmu.edu.tw; Tel.: +886-4-2205-3366-5302

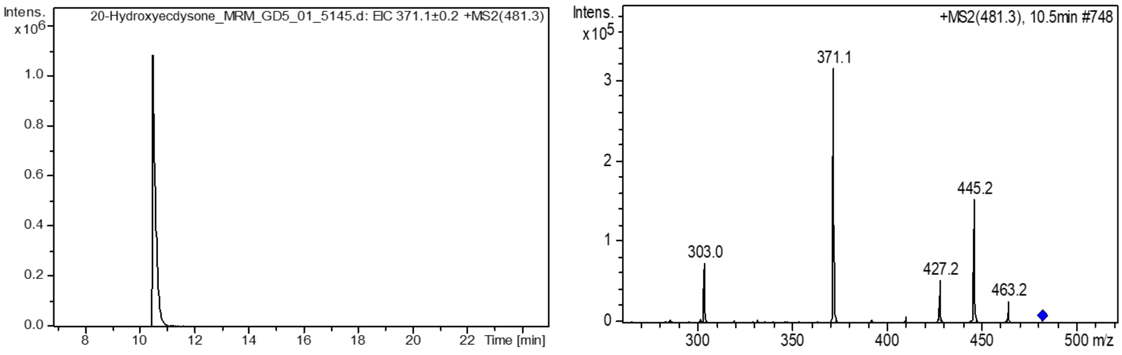

(**a**)

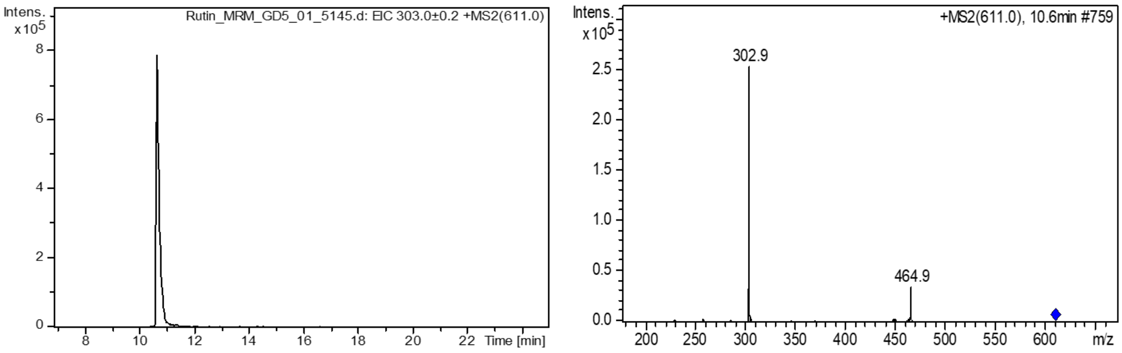

(**b**)

Figure S1. The multiple reaction monitoring (MRM) ion chromatogram and mass spectrum on electrospray ionization (ESI) positive mode of 20-hydroxyecdysone and rutin standard. **(a)** The transition from the precursor ion at *m*/*z* 481 to the product ion at *m*/*z* 371 for 20-hydroxyecdysone and **(b)** the transition from the precursor ion at *m*/*z* 611 to the product ion at *m*/*z* 303 for rutin.

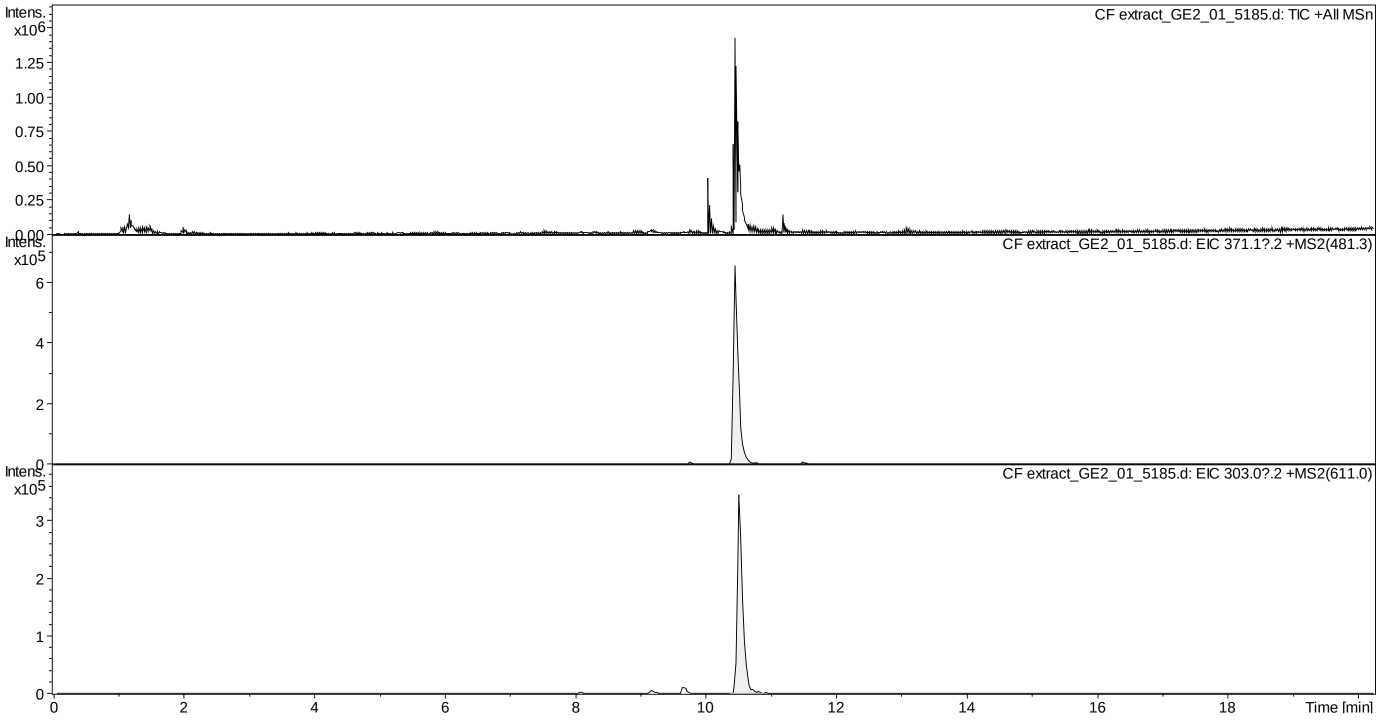

Figure S2. The total ion chromatogram (TIC) and extracted ion chromatogram (EIC) of 20-hydroxyecdysone (*m/z* 481) and rutin (*m/z* 611) in CF extract under MRM monitoring.
